# Antithrombotic Effects of Paeoniflorin from *Paeonia suffruticosa* by Selective Inhibition on Shear Stress-Induced Platelet Aggregation

**DOI:** 10.3390/ijms20205040

**Published:** 2019-10-11

**Authors:** Thien Ngo, Keunyoung Kim, Yiying Bian, Hakjun Noh, Kyung-Min Lim, Jin-Ho Chung, Ok-Nam Bae

**Affiliations:** 1 College of Pharmacy, Seoul National University, Seoul 08826, Korea; ngothien86@gmail.com (T.N.); millio1014@naver.com (K.K.); byy19900719@snu.ac.kr (Y.B.); hakjjun88@nate.com (H.N.); 2 College of Pharmacy, Ewha Womans University, Seoul 03760, Korea; 3 College of Pharmacy, Hanyang University, Ansan 15588, Korea; 4 Faculty of Pharmacy, Thai Binh University of Medicine and Pharmacy, Thai Binh city 410000, Vietnam; 5 School of Public Health, China Medical University, Shenyang 110122, China

**Keywords:** paeoniflorin, *Paeonia suffruticosa*, shear stress, antiplatelet, antithrombotic

## Abstract

Antiplatelet agents are important in the pharmacotherapeutic regime for many cardiovascular diseases, including thrombotic disorders. However, bleeding, the most serious adverse effect associated with current antiplatelet therapy, has led to many efforts to discover novel anti-platelet drugs without bleeding issues. Of note, shear stress-induced platelet aggregation (SIPA) is a promising target to overcome bleeding since SIPA happens only in pathological conditions. Accordingly, this study was carried out to discover antiplatelet agents selectively targeting SIPA. By screening various herbal extracts, *Paeonia suffruticosa* and its major bioactive constituent, paeoniflorin, were identified to have significant inhibitory effects against shear-induced aggregation in human platelets. The effects of paeoniflorin on intraplatelet calcium levels, platelet degranulation, and integrin activation in high shear stress conditions were evaluated by a range of in vitro experiments using human platelets. The inhibitory effect of paeoniflorin was determined to be highly selective against SIPA, through modulating von Willebrand Factor (vWF)-platelet glycoprotein Ib (GP Ib) interaction. The effects of paeoniflorin on platelet functions under high shear stress were confirmed in the ex vivo SIPA models in rats, showing the good accordance with the anti-SIPA effects on human platelets. Treatment with paeoniflorin significantly prevented arterial thrombosis in vivo from the dose of 10 mg/kg without prolonging bleeding time or blood clotting time in rats. Collectively, our results demonstrated that paeoniflorin can be a novel anti-platelet agent selectively targeting SIPA with an improved safety profile.

## 1. Introduction

Cardiovascular diseases (CVDs) are the leading cause of morbidity and mortality globally [1]. Hence, many efforts have been devoted to discovering novel therapeutic strategic, especially to prevent and cure thrombosis, one of the most serious complications of various CVDs [2]. Since platelets are crucial to thrombogenesis [3,4], platelet inhibitors, such as aspirin and clopidogrel, constitute a large component of CVDs pharmacotherapy [5]. These drugs have been proven their efficacy in preventing the recurrence of thrombosis [2,5]. However, bleeding, resulting from the disturbance of normal hemostasis, is still the most critical adverse [5]. In this context, it is urgent to discover novel anti-platelet agents selectively suppressing pathological thrombosis without influencing normal hemostasis [2].

Conventionally, thrombosis elicited by physiological agonists, such as thrombin, collagen, adenosine diphosphate (ADP), or arachidonic acid, has been a primary target for the development of platelet inhibitors [6,7]. Recently, a critical role of physical activator, i.e., high shear stress, is highlighted in the pathological thrombosis [8,9]. Distinct from platelet activation pathways dependent on physiological agonists, shear stress-induced platelet aggregation (SIPA) occurs solely in pathologic conditions, such as in stenotic vessels or atherosclerotic lesion [10,11]. In high shear stress conditions, the binding of von Willebrand factor (vWF) to glycoprotein (GP) Ib leads to signal transduction, thereby activating platelets [8]. When platelets are activated, the degranulation and GP IIb/IIIa activation on platelet surface further propagates, leading to the stabilization of platelet aggregates, and subsequent acceleration of thrombus formation [10,12]. In the pathogenic progress of thrombus formation, SIPA plays as a major contributor [8]. SIPA becomes one of the promising targets of new antiplatelet agents that could overcome the bleeding issue of conventional platelet inhibitors [2,13].

Natural products are great sources of medicines [14]. With the aim of discovering the highly potent and safe antiplatelet drugs, we introduced shear stress as a platelet stimulator to explore the effects of diverse herbal extracts which are used as traditional CVDs remedies. We identified the extract of the dried root bark of *Paeonia suffruticosa* Andrew (Ranunculaceae/Paeoniaceae) and its active ingredient, paeoniflorin, has strong activities against SIPA. We also investigated the in vivo efficacy of paeoniflorin against thrombosis along with its potential adverse effects in rat models.

## 2. Results

### 2.1. Paeoniflorin from Paeonia Suffruticosa Extract Significantly Inhibited Shear Stress-Induced Platelet Aggregation

**Figure 1:**
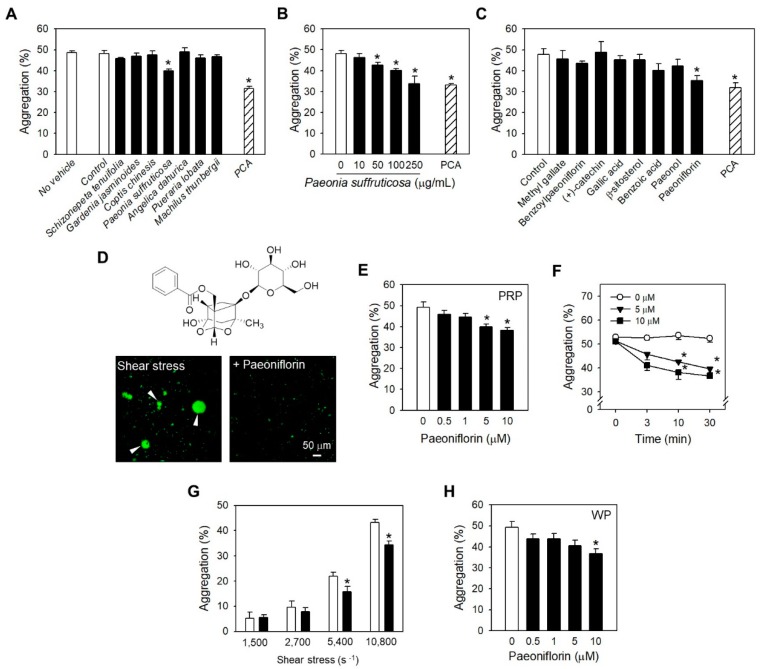
Inhibitory effects of *Paeonia suffruticosa* extract and paeoniflorin on shear stress-induced platelet aggregation (SIPA). (**A**) Effects of herbal extracts (100 µg/mL) on SIPA in human platelet-rich-plasma (PRP) (*n* = 3). (**B**) Concentration-dependent effects of *Paeonia suffruticosa* extract on SIPA (*n* = 3). (**C**) Effects of the active ingredients of *Paeonia suffruticosa* (25 µM) on SIPA in PRP (*n* = 3). Insert, the molecular structure of paeoniflorin. Protocatechuic acid (PCA, 25 µM) was used as positive control. (**D**) Molecular structure (upper) and fluorescent observation inhibitory effect of paeoniflorin platelet aggregation in pathological shear stress (lower). Green: Calcein-loaded platelets. (**E**–**F**), Inhibitory effects of paeoniflorin on SIPA in human PRP, in a concentration- (10 min treatment) (*n* = 5) (**E**) and time-dependent manner (*n* = 3) (**F**). (**G**) Effects of paeoniflorin on SIPA at different levels of shear rate (*n* = 3), (**H**). SIPA inhibition by paeoniflorin in human washed platelets (*n* = 4). Values are mean ± standard error of the mean (SEM; expressed as the error bar) of the independent experiments from different blood donors. *, significant differences from control group (*p* < 0.05). Arrowhead indicated platelet aggregates (**D**). Scale bar: 50 μm.

The extracts of Schizonepeta tenuifolia, Gardenia jasminoides, Coptis chinesis, *Paeonia suffruticosa*, *Angelica dahurica*, *Pueraria lobate*, and *Machilus thunbergii*, which are well-known as traditional remedies for cardiovascular diseases [15,16,17,18,19,20], were employed for investigating their activities against high shear stress-induced platelet aggregation (SIPA). Among the tested herbal extracts, *Paeonia suffruticosa* (*P. suffruticosa*) extract showed the most potent efficacy (Figure 1A). The inhibitory effect of the *P. suffruticosa* extract on SIPA was in a concentration-dependent manner (Figure 1B). In order to identify the active components of *P. suffruticosa*, which are accountable for SIPA inhibition, eight major ingredients of *P. suffruticosa* were examined (25 µM, Figure 1C). As a result, paeoniflorin exhibited the highest efficacy among all tested compounds with statistical significance (Figure 1C). Microscopic observation also showed a remarkable reduction in the number and the size of platelet aggregates following the treatment with paeoniflorin (Figure 1D). The effects of paeoniflorin were in concentration- (Figure 1E) and time-dependent manners (Figure 1F). The effectiveness of paeoniflorin in suppressing platelet aggregationinduced by shear stress was investigated with the various levels of shear rates, which can be found at different rheological conditions (Figure 1G). As a result, at high levels of shear rate, 5400 and 10,800 s^−1^, paeoniflorin at 25 µM significantly prevented platelet aggregation due to shear stress (Figure 1G). Inhibition of SIPA by paeoniflorin was further confirmed in human washed platelets (Figure 1H), wherein the participation of plasma protein was excluded. These data reflected that anti-SIPA effect of paeoniflorin is targeted on platelets.

### 2.2. Paeoniflorin Selectively Inhibits Platelet Aggregation-Induced by Shear Stress

**Figure 2:**
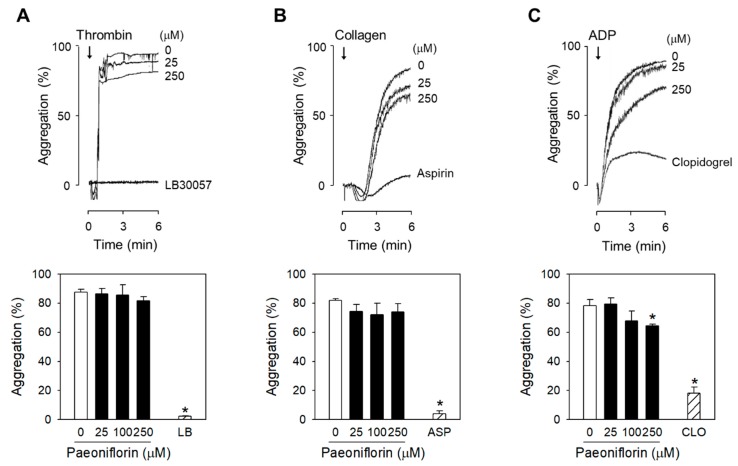
Selective effects of paeoniflorin on SIPA. (**A**–**C**) Various concentrations of paeoniflorin were treated with human PRP for 10 min, and then stimulated by thrombin (0.6–0.8 U/mL) (**A**), collagen (2–4 μg/mL) (**B**), or ADP (5–10 μM) (**C**), respectively (*n* = 3). The platelet aggregation was measured by turbidimetric aggregometer. LB30057 (LB, thrombin inhibitor), aspirin (ASP) or clopidogrel (CLO) was used as positive control of each assay, respectively. Values are mean ± SEM (expressed as the error bar) of the independent experiments from different blood donors.

The inhibition of platelet functions of paeoniflorin was further evaluated with the stimulation of platelet endogenous agonists, including thrombin, collagen, and ADP. Interestingly, treatment with the high concentrations of paeoniflorin, up to 250 µM, the platelet aggregation was not affected following stimulation by thrombin (Figure 2A) or collagen (Figure 2B). In ADP-stimulated platelets, statistically significant inhibition of aggregation was only found in high concentration of paeoniflorin at 250 µM (Figure 2C). Meanwhile, paeoniflorin significantly inhibited shear-induced platelet aggregation (Figure 1E) from the concentration of 5 µM, suggesting that the effect of paeoniflorin is highly selective against the pathological SIPA.

### 2.3. Paeoniflorin Inhibits Platelet Activation Events in High Shear Stress Condition

**Figure 3:**
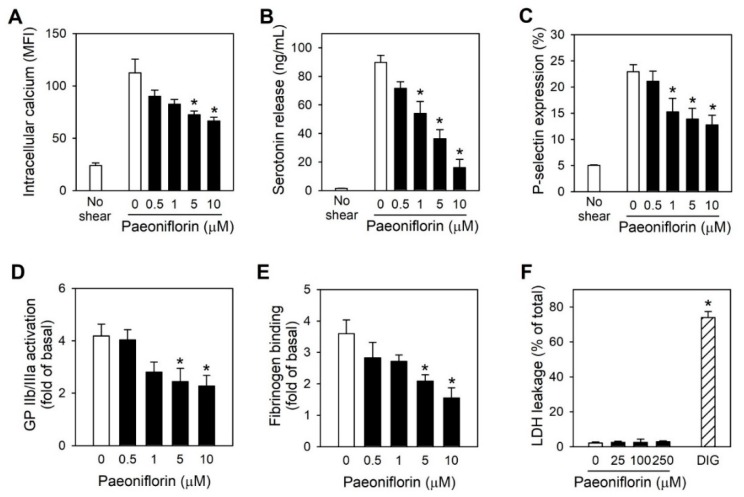
Effects of paeoniflorin on platelet activation events-triggered by shear stress. Human washed platelets were treated with different concentrations of paeoniflorin for 10 min before exposing to high shear stress. The intraplatelet signaling was investigated, including cytoplasmic calcium level was measured by using Fluo-4AM-loaded platelets (**A**) (*n* = 4), serotonin release (*n* = 4) was measured by ELISA (**B**), P-selectin expression (**C**) (*n* = 4), GP IIb/IIIa activation (**D**) (*n* = 4), and fibrinogen binding (*n* = 4) (**E**) were measured by flow cytometry. (**F**) Cytotoxicity induced by paeoniflorin on platelets was measured by LDH leakage. (DIG, Digitonin 50 μM treatment for 1 h was used as positive control) (*n* = 3). Values are mean ± SEM (expressed as the error bar) of the independent experiments from different blood donors. *, significant differences from control group (*p* < 0.05).

Intracellular calcium levels are known to be a key player in platelet activation and aggregation [21], the change of intraplatelet calcium levels under high shear stress was investigated following by the treatment with paeoniflorin. Paeoniflorin significantly prevented the elevation of cytosolic calcium levels-induced by shear stress in a concentration-dependent manner (Figure 3A). Upon activation, platelets secrete large amounts of mediators, which can further propagate platelet activation and aggregation progress, facilitating thrombus formation and cardiovascular diseases [22]. Treatment with paeoniflorin effectively suppressed shear stress-induced platelet degranulation from both dense and α-granule, as examined by serotonin release and P-selectin expression (Figure 3B,C). Furthermore, the effects of paeoniflorin on GP IIb/IIIa activation and fibrinogen binding, which are important for stabilizing platelet aggregates [11] were also determined. Paeoniflorin inhibited shear stress-induced GP IIb/ IIIa activation and fibrinogen binding in a concentration-dependent manner (Figure 3D,E). Of note, these effects of paeoniflorin on platelet activity were not associated with cytotoxicity as no increase in LDH leakage was shown in the investigation up to concentration of 250 µM of paeoniflorin (Figure 3F).

### 2.4. Paeoniflorin Inhibited SIPA through Modulating vWF-GP Ib Interaction

**Figure 4:**
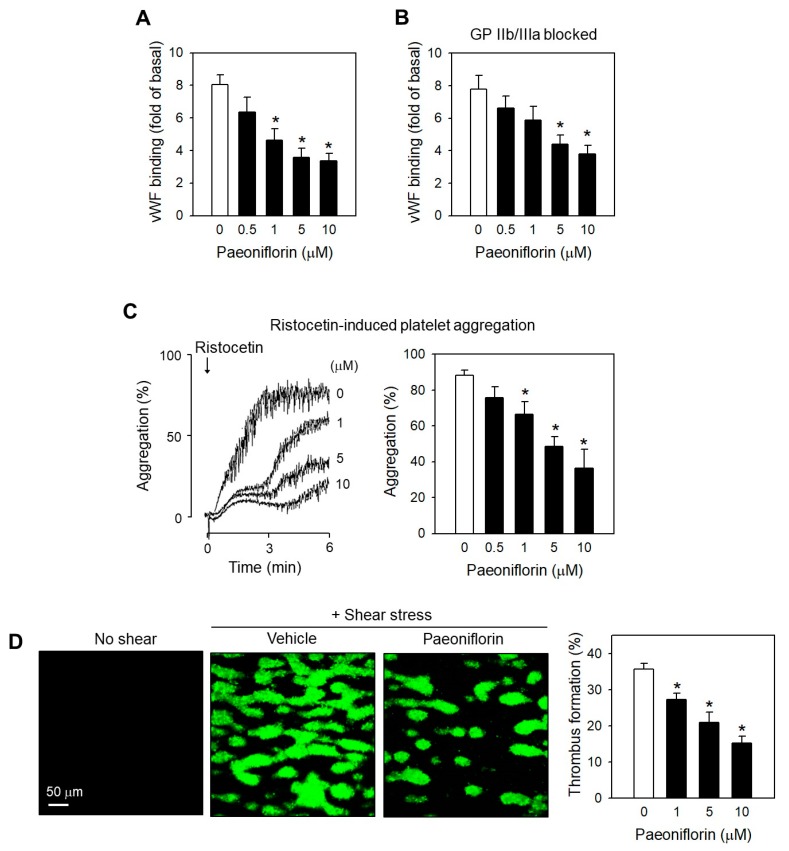
Effects of paeoniflorin on SIPA depending GPIb-vWF interaction. (**A**,**B**) Effects of paeoniflorin on vWF binding to platelets under high shear stress was determined by flow cytometry, in the absence (*n* = 4) (**A**) and in the presence of GPIIb/IIIa blocker (*n* = 4) (**B**). Arg-Gly-Asp (2 mM) was used to block GPIIb/IIIa by pre-incubated with platelets. **C**, Effects of paeoniflorin on ristocetin-induced platelet aggregation in human PRP (*n* = 4). (**D**) Effects of paeoniflorin on platelet adhesion on collagen (100 µg/mL) coated surface under shear stress (1500 s^−1^ for 5 min) in flow chamber (*n* = 3). The platelet adhesion was observed by confocal microscopy LSM-710 and the coverage area was analyzed by ImageJ. Values are mean ± SEM (expressed as the error bar) of the independent experiments from different blood donors. *, significant differences from control group (*p* < 0.05). Scale bar (**D**): 50 μm. Green: Calcein-loaded platelets.

Platelet activation/ aggregation depending on high shear stress is initiated by the interaction of the von Willebrand factor (vWF) with glycoprotein (GP) Ib on the platelet surface [23]. Therefore, we further investigated the effects of paeoniflorin on this interaction. Interestingly, the engagement of vWF to platelets under shear stress was remarkably attenuated following by treatment with paeoniflorin in a concentration-related manner (Figure 4A). The inhibitory effects of paeoniflorin persisted even though GP IIb/IIIa, another binding site of vWF in platelet membrane, was blocked (Figure 4B), suggesting the effects of paeoniflorin primarily interfering vWF-GP Ib interaction. This inhibitory effect of paeoniflorin was further confirmed in the platelet aggregation assay induced by ristocetin (Figure 4C), an exogenous platelet activator inducing vWF and GPIb interaction in the static condition which is close to the effects of high shear stress [24]. The modulatory effects of paeoniflorin on platelet functions under shear stress were further evaluated in flow chamber with collagen-coated surface. Paeoniflorin treatment effectively inhibited thrombus formation in a concentration-dependent manner (Figure 4D).

### 2.5. Paeoniflorin Significantly Prevented Thrombus Formation without Increased Bleeding Risk

**Figure 5:**
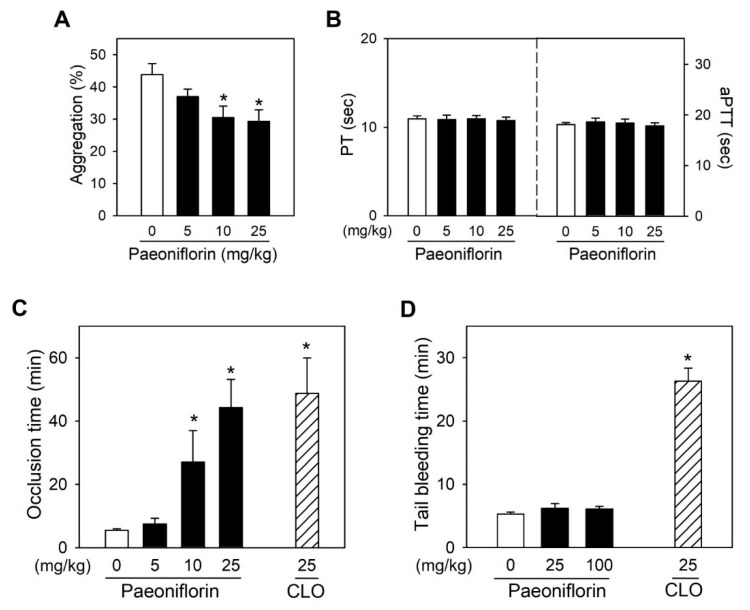
In vivo effects of paeoniflorin on arterial thrombosis and bleeding time. (**A**) Ex vivo effects of paeoniflorin on SIPA in freshly isolated rat platelets, which were collected from SD rats following the intravenous treatment with paeoniflorin for 30 min (*n* = 5). (**B**) In vivo effects of paeoniflorin on thrombin time (PT), and activated partial prothrombin time (aPTT) (*n* = 5). (**C**) In vivo effects of paeoniflorin on time to occlusion the in rat arterial thrombosis model induced by FeCl_3_ (*n* = 5). (**D**) Effects of paeoniflorin on bleeding time in rat tail transection model. The in vivo efficacy of paeoniflorin was compared to that of clopidogrel (CLO) at the dose of 25 mg/kg by oral administration (*n* = 5). Values are mean ± SEM (expressed as the error bar) of at the independent experiments from different animals. *, significant differences from control group (*p* < 0.05).

Ex vivo SIPA experiments in SD rats following intravenous administration of paeoniflorin was used to determine the differences in species response of platelets under high shear stress. As a result, treatment with paeoniflorin significantly inhibited high shear stress-dependent platelet aggregation depending on doses, in a good agreement with in vitro results in human platelets (Figure 5A), strongly supporting the in vivo efficacy of paeoniflorin on platelet activity both in two species of humans and rats. Notably, at these dose regimens inhibiting SIPA in rat platelets, paeoniflorin did not influence blood clotting time, as determined with prothrombin time (PT) and activated partial thromboplastin time (aPTT) (Figure 5B). Treatment with paeoniflorin significantly prevented ferric chloride-induced arterial thrombosis (Figure 5C), where platelets play a major role [25]. Pre-treatment with paeoniflorin with the dose of 5, 10, and 25 mg/kg prolonged the occlusion time to 7.5 ± 1.8 min, 27.1 ± 9.9 min, and 44.4 ± 8.9 min, respectively). Meanwhile, the occlusion time in vehicle-treated rats was 5.5 ± 0.5 min. Of note, while clopidogrel, one of the most frequently used antiplatelet drug [2], increased bleeding time at the effective anti-thrombotic dose (Figure 5C,D), paeoniflorin did not affect the bleeding time even at the dose of ten times higher than anti-thrombotic dose (Figure 5D), highlighting the antiplatelet effects of paeoniflorin with a favorable safety profile.

## 3. Discussion

This study demonstrated the antiplatelet effects of *P. suffruticosa* extract and its bioactive constituent, paeoniflorin. The mechanism of antithrombotic effects by paeoniflorin was elucidated to selectively inhibit shear stress-dependent platelet aggregation. The effects of paeoniflorin on platelet activation were mediated by blocking vWF-platelet GP Ib interaction. Antithrombotic effect of paeoniflorin was confirmed in vivo without a significant increase in bleeding risk, supporting that paeoniflorin may be a promising platelet inhibitor from natural products with a safety profile.

Natural products have been an important resource for developing novel medications, in particular, cardiovascular remedies [26,27]. Herbal extracts or the bioactive constituents have been widely investigated for their activities in modulating platelet functions, especially platelet activation induced by endogenous agonists including thrombin, collagen, ADP or prostaglandin, thromboxane A2 [28,29]. Aspirin, discovered first in the willow bark (*Salix alba vulgaris*) [30], is now one of the common platelet inhibitors used in clinical [2]. Unfortunately, the elevated gastrointestinal bleeding incidence was recorded to be associated with aspirin therapy [31] and many traditional herbal medicines are not also free from bleeding risks [32,33]. This is ascribable to that these drugs are discovered based on the inhibitory activities against platelet activation induced by endogenous agonists, which is commonly critical in suppressing bleeding. In this context, we screened a wide range of herbal extracts focusing on the pathological shear stress, which may overcome bleeding complications [2,34].

*P. suffruticosa*, with the traditional name, moutan cortex, has been used as remedies for improving blood circulation in cardiovascular diseases, amenorrhea, dysmenorrhea, and pain in the chest and abdomen [15]. Here, the modulatory effect of *P. suffruticosa* on platelet functions through SIPA inhibition was described (Figure 1A,B), to which paeoniflorin, its monoterpene glucoside component, made the greatest contribution (Figure 1C). Of note, paeonol, another bioactive ingredient of moutan cortex, which is generally believed as the major contributor for the antithrombotic effects [15], did not exhibit a significant effect (Figure 1C). Paeoniflorin has been recognized for its influence on thrombosis [35]. The early report by Ye et al. suggested the inhibition of arachidonic acid metabolism by paeoniflorin, including suppression on prostacyclin production in endothelial cells and thromboxane A_2_ in platelets [35]. We have demonstrated that the effects of paeoniflorin on platelets is highly selective to shear-induced activation (Figure 2), suggesting shear specific event such as vWF-GP Ib binding would be more essential mechanism in antiplatelet activity of paeoniflorin. Interestingly, in higher concentration of paeoniflorin (250 μM), ADP-induced platelet aggregation was significantly inhibited, reflecting that ADP-P2Y12 activation may also be modulated by paeoniflorin. Orchestrated effects on SIPA specific events and/or TXA_2_ or P2Y12 activation may enhance the potential in vivo effectiveness of paeoniflorin, which may support the therapeutic application of P. suffruticosa for the prevention of thrombosis.

The activation pathways in platelets are mainly initiated by the interactions between physiological agonists including thrombin, collagen and ADP, and their representative receptors [22,36]. Similar to the initiation of platelet activation by these agonists, shear stress promotes the integration of vWF to its ligand on the platelet surface, glycoprotein GP Ib [13]. The interaction of vWF and GP Ib subsequently initiates common intracellular signals leading to platelet activation [22]. These include protein tyrosine phosphorylation, activation of protein kinase C, activation of phosphoinositide 3-kinase, elevation of the intracellular calcium [37]. Paeoniflorin modulated the essential platelet activation events including intraplatelet calcium level increase (Figure 3A) and platelet degranulation, both α-granules (as detected by P-selectin, Figure 3B) and dense granules (as detected by serotonin release, Figure 3C). Not only these intraplatelet activation pathways, paeoniflorin also regulated the inside-out signaling pathways which evoke platelet-platelet interactions or other tissues to amplify platelet activation [10,38,39], as demonstrated by suppression of the activation of GP IIb/IIIa (Figure 3D), and the reduced binding of fibrinogen to the activated integrin (Figure 3E). Since activated platelets can contribute to amplify the aggregation and coagulation processes by thrombin generation or exposure of phosphatidylserine [40], further study is warranted to investigate if paeoniflorin affects these changes to enhance its potential for clinical intervention.

While paeoniflorin significantly inhibited the change of platelet activation markers including intraplatelet calcium level and platelet degranulation (Figure 3), the inhibitory effect of paeoniflorin was found to be selective to shear-induced platelet activation (Figure 2). This suggests that the influence of paeoniflorin on the integration of vWF and GP Ib (Figure 4A,B) may be critical for the modulation of subsequent platelet activation events (Figure 3). The interaction between vWF and GP Ib under hydrodynamic activation is mediated by complicated mechano-regulated processes [41]. Several hypotheses might explain the inhibitory effect of paeoniflorin on vWF-GP Ib interaction, (1) direct interaction with vWF, (2) interfering shear-induced transformational or functional change of vWF, (3) modulation of GP Ib function, and (4) interfering the binding between vWF A1 domain and GP Ib α. In this study, we have focused on the selective inhibitory activity of paeoniflorin on shear-induced platelet activation in vitro and in vivo models, but the biochemical elucidation of the precise mechanisms of paeoniflorin action on vWF-GP Ib interaction would be required to enhance its potential as a therapeutic option for thrombotic diseases.

Since the conventional turbidimetric method to determine platelet aggregation is not applicable to SIPA, platelet aggregation in this study was measured by a particle counting method [34,42]. This method is proved to be useful for quantitative platelet aggregation compared to the turbidimetric aggregometry [43], in terms that single platelet counting is sensitive to microaggregation, whereas turbidimetric aggregometry (light-transmission aggregometry) measures macroaggregation [44]. Using optical particle counting, the singlet platelets can be distinguished from the triplets or quadruplets [43]. The main disadvantages of these methods are that it is not suitable for measurement of the events occurring in less than 500 msec [43]. In this study, the loss of single platelets after exposing to high shear stress for 10 min was measured by microscopic particle counting with hematocytometer. We did not require the kinetic platelet aggregation by shear stress, therefore our data were not affected by the limitation of the method.

In the line with in vitro results revealing the antiplatelet effects of paeoniflorin, the thrombus formation in arterial shear stress in a flow chamber was significantly suppressed by treatment with paeoniflorin (Figure 4D). The potent antithrombotic efficacy of paeoniflorin was confirmed in vivo in arterial thrombosis models (Figure 5C). The selective effects of paeoniflorin on pathological platelet aggregation dependent on shear stress was illustrated in the in vivo study as paeoniflorin effectively prevented thrombosis without inducing blood clotting time nor bleeding time (Figure 5B–D). Notably, at the dose ten times higher than the antithrombotic effective dose, paeoniflorin did not increase hemorrhage risk (Figure 5D). These results strongly support that paeoniflorin may have a good efficacy and safety profile for an antiplatelet agent for the prevention of cardiovascular disorders.

In this study, rats were given paeoniflorin at doses of 5, 10, and 25 mg/kg intravenously, and the arterial thrombosis was induced at 30 min after treatment. Although we have not measured the blood level of paeoniflorin in the current experiment, the time point and the dose ranges might be suitable for the possible distribution of paeoniflorin in blood and platelets. In previous literature by Wang et al. [45] regarding paeoniflorin kinetics in rats, intravenous injection of paeoniflorin at 30 mg/kg showed the two-compartment model of distribution, with t_1/2α_ at 30 min. Moreover, Taketa et al. [46] demonstrated that in the plasma concentration-time curve of paeoniflorin after intravenous administration for 30 min with a dose of 5 mg/kg, the plasma concentration was estimated of 2 µM. The dose range of 5, 10 and 25 mg/kg might lead micromolar plasma concentration (>2 µM), which could be in the similar range with the effective concentration in our in vitro results for significant inhibitory effects on platelet activity under high shear stress (Figure 1, Figure 2, Figure 3 and Figure 4).

The implication of shear-driven platelet activation has been increased as it can be an important contributor to thrombotic complications under “high shear” condition, which might be naturally found in stenotic regions, or is artefactually produced in abnormal flow patterns by implantable cardiovascular therapeutic devices. In arterial flows, shear stress is in the range of 0–30 dynes/cm^2^ [8]. However, it may elevate to 300 dynes/cm^2^ in stenosis areas [47], and even exceed 1000 dynes/cm^2^, exerted by therapeutic mechanical cardiovascular blood recirculating devices [48]. These supra physiological levels (hypershear) happen in mechanical circulatory support (MCS) devices such as ventricular assist devices (VADs), total artificial heart (TAH), and prosthetic heart valves. Interestingly, under these conditions, platelet activation can occur directly in the free flow, regardless of the participation of vWF or endothelium [48]. In this study, we have focused on investigating the impact of paeoniflorin on shear stress-induced platelet aggregation in pathological conditions, which is mainly mediated by vWF-GP Ib interactions [13,38]. As urgent need exists for anti-thrombotic pharmacotherapy for safe use of VAD or TAH, it will be worthy to investigate the effects of paeoniflorin on recently established hypershear-mediated platelet activation models to expand the therapeutic scope.

## 4. Materials and Methods

### 4.1. Materials

Paeoniflorin, adenosine diphosphate (ADP), trisodium citrate, HEPES, prostaglandin E_1_ (PGE_1_), glutaraldehyde, EDTA, EGTA, Tris-base, Tris-HCl, β-NADH, pyruvic acid, Triton X-100, Arg-Gly-Asp, ferric chloride, urethane, clopidogrel, and bovine serum albumin were obtained from Sigma-Aldrich (St. Louis, MO, USA). Thrombin was obtained from Calbiochem (San Diego, CA, USA). Collagen and ristocetin were from Chrono-log (Harvertown, PA, USA). vWF was from Molecular Innovations, Inc. (Nove, MI, USA). Fluo-4/AM, pluronic F-127, and alexa fluor 488-conjugated fibrinogen were from Invitrogen (Carlsbad, CA, USA). Phycoerythrin (PE)-labeled monoclonal antibody against human CD42b (antiCD42b-PE Ab), Fluorescein isothiocyanate (FITC)-labeled anti-CD62P antibody (anti-CD62P-FITC Ab), and FITC-labeled PAC-1 (PAC-1-FITC) were from BD Biosciences (San Jose, CA, USA), and FITC-labeled anti-vWF antibody (anti-vWF-FITC) was from Abcam plc (Cambridge, UK). All the reagents were used with the highest available purity.

The extracts of *Schizonepeta tenuifolia*, *Gardenia jasminoides*, *Coptis chinesis*, *Paeonia suffruticosa* (*P. suffruticosa*), *Angelica dahurica*, *Pueraria lobate*, and *Machilus thunbergii*, and main herbal constituents from P. suffruticosa dried rout bark (moutan cortex) were supplied by the Natural Medicine Research Institute of Seoul National University.

The powdered moutan cortex was refluxed with 70% EtOH for 3 h at 70–80 °C. The ethanol extract was dried by evaporating under low pressure. The ethanol extract of Moutan Cortex was suspended in H_2_O and partitioned with n-hexane, CH_2_Cl_2_, EtOAc, and n-BuOH. The ethyl acetate layers were combined and evaporated under vacuum. This fraction was loaded on a silica gel column and eluted with a gradient of CH_2_Cl_2_/MeOH/H_2_O (70: 8: 5, 70: 10: 5, 70: 16: 5, 70: 20: 5, 7: 3: 1, and 13: 7: 2) to give 16 fractions (F1-F16). Fraction F5 was chromatographed on a silica gel column and eluted with a gradient of n-hexane/ EtOAc (75: 25 to 100% in 5% steps) to obtain 6 sub-fractions (F5.1–F5.6). Subfraction F5.3 was further purified using an RP-18 column with 50% MeOH/water to yield paeoniflorin. Paeoniflorin isolated by this method was used for Figure 1A,B. For other in vitro and in vivo results, standard paeoniflorin purchased from Sigma-Aldrich (St. Louis, MO, USA) was used to secure the amount and ensure the quality of the material. We confirmed that isolated and commercial paeoniflorin showed similar activities against SIPA. For in vitro experiments, the herbal extract and tested compounds were dissolved in DMSO (final, 0.5%). Vehicle (DMSO 0.5%) did not alter platelet aggregation level. In terms of in vivo experiments, paeoniflorin was dissolved in normal saline.

### 4.2. Human Platelet Preparation

With the approval from the Institutional Review Board of Seoul National University (IRB No. 1702/003-004, 5, March, 2019),), whole blood was collected from non-smoking healthy male volunteers (18–25 years old) on the day of experiments. To prevent the activation of platelets during isolation procedure, usage of glass and strong mechanical forces were avoided. For platelet-rich-plasma (PRP) preparation, whole blood with 3.2% trisodium citrate was centrifuged at 150× *g* for 15 min. The top layer was carefully transferred as PRP. The remaining fraction (buffy coat and red blood cell layer) was centrifugation at 2000 *g* for 20 min to obtain platelet-poor-plasma (PPP). Platelet cell count in PRP was adjusted 3 × 10^8^ platelets/mL by diluting with PPP. For washed platelet (WPs) preparation, whole blood was carefully collected with anti-coagulant of acid citrate-dextrose (ACD, 85 mM trisodium citrate, 71 mM citric acid, and 111 mM glucose, 1: 6, *v*/*v*), supplemented with PGE_1_ 1 μM. PRP was prepared as described above and was further centrifuged for 10 min at 500 *g* to obtain platelet pellets. The platelet pellets were carefully washed with calcium-free Tyrode′s buffer (134 mM NaCl, 2.9 mM KCl, 1.0 mM MgCl_2_, 10.0 mM HEPES, 5.0 mM glucose, 12.0 mM NaHCO_3_, 0.34 mM Na_2_HPO_4_, and 0.3% bovine serum albumin, pH 7.4) containing 10% ACD and PGE_1_ 1 μM. After centrifugation at 400 *g* for 10 min, platelets were finally suspended at 3 × 10^8^ platelets/mL in Tyrode′s buffer containing 2 mM CaCl_2_, as described previously [34].

### 4.3. Measurement of Shear Stress-Induced Platelet Aggregation

Platelet suspension was exposed to the shear rate at 10,800 s^−1^ for 10 min at 37 °C by using a programmable cone-plate viscometer (RotoVisco 1, Thermo Fisher Scientific, Waltham, MA, USA). Human platelets were treated with tested compounds (in DMSO, final 0.5%) or vehicle (DMSO) for 10 min. For WPs, before applying shear stress, 10 µg/mL of vWF was added. The shear stress exposed platelets were fixed by 0.5% glutaraldehyde in Tyrode′s buffer, to block further activation or aggregation during platelet count. The extent of platelet aggregation was evaluated based on the loss of single cell as in comparison to the non-sheared sample, as described previously with a minor modification [34], according to the number of single platelets per microliter under a phase-contrast light microscope (CX41, Olympus, Tokyo, Japan). Prior to counting, platelet suspensions were diluted to approximately 300 to 500 particles in 5/25 squares. The variations between two different fixed samples from the same platelet suspension were typically <3% from the mean.

For visualization platelet aggregation induced by shear stress, human PRP was stained with calcein-AM (5 µM) in dark for 30 min. The samples were treated with paeoniflorin or vehicle before subjected to shear stress. The collected samples were fixed by paraformaldehyde 2% for 10 min before spreading on slide glass for imagined by confocal microscopy LSM-710.

### 4.4. Assessment of Cytoplasmic Calcium Levels

To determine intraplatelet calcium levels, washed platelets were loaded with fluo-4/AM (5 µM) and pluronic F-127 (0.2%) for 45 min at 37 °C in the dark. The treated platelets with paeoniflorin for 10 min at 37 °C were exposed to shear stress, then diluted by Tyrode’s buffer. The change of cytosolic calcium levels was analysed on the FACS Calibur cytometer (BD Biosciences, Franklin Lakes, NJ, USA) equipped with an argon laser (λ_ex_ 488 nm). Data from 10,000 events were collected and analysed by using CellQuest Pro software (BD Biosciences, Franklin Lakes, NJ, USA).

### 4.5. Assessment of P-Selectin Expression, GP IIb/IIIa Activation, and Fibrinogen Binding

The antibody CD62P-FITC Ab, PAC-1-FITC, alexa fluor 488-conjugated fibrinogen, or anti-vWF-FITC Ab was used as the marker to determine P-selectin expression, glycoprotein (GP) IIb/IIIa activation, fibrinogen binding, or vWF binding, respectively. Anti-CD42b-PE Ab was used to identify platelets. Washed platelets were treated with paeoniflorin for 10 min at 37 °C before exposing to shear stress at 18,000 s^−1^ for 3 min. Sheared platelets were collected and incubated with the mixture of antibodies for 20 min in the dark, at room temperature. Platelets were analyzed on flow cytometry was described above. In some experiments, Arg-Gly-Asp 2 mM was used to block glycoprotein IIb/IIIa (GP IIb/IIIa).

### 4.6. Assessment of Platelet Cytotoxicity

Leakage of lactate dehydrogenase (LDH) from platelets was measured by spectrophotometric analysis as described previously with minor modification [34]. After incubation with paeoniflorin for 10 min at 37 °C, the supernatant obtained from the centrifugation reaction mixture was used in the LDH assay. The extent of cell lysis was expressed as the percentage of total enzyme activity compared to that of a control incubation lysed with 0.3% Triton X-100.

### 4.7. Measurement of Agonist-Induced Platelet Aggregation in human PRP

To evaluate platelet aggregation dependent on physiological agonists was used. PRP was treated with paeoniflorin for 10 min at 37 °C and then loaded on the aggregometer. Platelet aggregation was stimulated by adding thrombin (0.6–0.8 U/mL), collagen (2–4 µg/mL), ADP (5–10 µM), or ristocetin (1.0–1.25 mg/mL), respectively for 6 min. Platelet aggregation was measured by light transmission, with 100% calibrated as the absorbance of PPP.

### 4.8. Determination Effects on Thrombus Formation under Flow Conditions

To evaluate the in vitro thrombus formation, flow chambers (microslide VI^0.1^, iBidi) were coated with 100 µg/mL collagen (type I, Chrono-log) for 60 min at room temperature. After washing with phosphate-buffered saline (PBS) for 3 times, the chambers were blocked by 1% bovine serum albumin/ PBS for 60 min. Whole blood, loaded with calcein AM 5 µM, was treated with paeoniflorin for 10 min, then was perfused to coated flow chambers at a shear rate of 1500 s^−1^ for 5 min by syringe pump. The thrombus formation was imagined by confocal microscopy LSM-710 and the data was analyzed by ImageJ on three random fields.

### 4.9. Determination of Serotonin Release

WPs were treated with paeoniflorin for 10 min at 37 °C before subjected to shear stress at 10,800 s^−1^ for 3 min. The reactions were terminated by adding EDTA 5 mM. The resulting aliquot obtained by centrifugation at 12,000 rpm for 5 min at 4 °C underwent serotonin detection by serotonin ELISA kit (Labor Diagnostika Nord GmbH & Co., Nordhorn, Germany) according to manufacturer’s instructions.

### 4.10. Ex Vivo Determination of Anti-SIPA Activity

All the protocols were approvedby the Institutional Animal Care and Use Committee of the Animal Service Center at Seoul National University (IACUC No. SNU-170417-27-4, 23/01/2019). Male Sprague-Dawley (SD) rats (SamTako Co., Osan, Korea), weighing 280–320 g, used in all experiments, were acclimated for 1 week. Food and water were provided ad libitum.

For the measurement of ex vivo shear-induced platelet aggregation, 30 min after paeoniflorin or vehicle (normal saline) was administered intravenously. Whole blood was collected from abdominal aorta under anesthesia by using an anticoagulant of 3.8% trisodium citrate (1:9 citrate/blood, *v*/*v*). PRP was prepared and effects on SIPA were determined as described above. The isolated plasma was used for determination prothrombin time (PT) and activated partial thromboplastin time (aPTT) as per the instruction of the manufacturer.

### 4.11. In Vivo Arterial Thrombosis Model

To evaluate the effects of paeoniflorin on thrombosis formation in arteries, the model of FeCl_3_-induced arterial thrombosis was used. After intravenous administration of paeoniflorin or vehicle (normal saline), rats were anesthetized with urethane (1.25 g/kg, i.p.) and approximately 15 mm of the right artery was exposed and dissected free of nerve and connective tissue. Filter paper (1 × 2 mm, Whatman, Clifton, NJ, USA) which was soaked with 50% FeCl_3_, was applied to the carotid artery for 10 min. A Doppler flowmeter probe (Transonic Systems Inc., Ithaca, NY, USA) was placed around the arterial segment proximal to the injured site to assess blood flow. The time to occlusion was measured for up to 60 min.

### 4.12. In Vivo Tail Bleeding Time Measurement

To determine the effects of paeoniflorin on bleeding risk, the transection tail bleeding model in rats was introduced. After 30 min of paeoniflorin application or vehicle (normal saline), the rat tails were transected at a site 3 mm proximal to the tip. Filter paper (Whatman, Clifton, NJ, USA) was used to gently blot the blood flowing from the incision every 30 s. Bleeding time was measured as time elapse until bleeding stopped. If after 30 min, the blood still bled from the injured site, measurement was stopped, and bleeding time was recorded as 30 min.

### 4.13. Statistical Analysis

All data are shown as mean ± SEM, and were subjected to one-way analysis of variance followed by Duncan’s multiple ranged tests to determine which means were significantly different from the control. Statistical analysis was performed with SPSS software (SPSS Inc., Chicago, IL, USA). In all cases, *p* < 0.05 was used to determine significance.

## 5. Conclusions

In conclusion, we demonstrated that *P. suffruticosa* extract and paeoniflorin have potent antiplatelet effects through the selective inhibition of SIPA. Our results suggested that paeoniflorin could be a promising novel antiplatelet agent to prevent thrombotic complications with strong potency and a wider margin of safety profile in terms of bleeding, which warrants further study in the future.

## Abbreviations

Ab	antibody
ADP	adenosine diphosphate
AM	acetoxymethyl ester
aPTT	activated partial thromboplastin time
CLO	clopidogrel
DMSO	dimethyl sulfoxide
FITC	fluorescein isothiocyanate
GP	glycoprotein
LDH	lactate dehydrogenase
PBS	phosphate-buffered saline
PE	phycoerythrin
PF	paeoniflorin
PGE_1_	prostaglandin E_1_
PRP	platelet-rich plasma
WP	washed platelet
PT	prothrombin time
SIPA	shear stress-induced platelet aggregation
vWF	von Willebrand factor
